# A call to action for blood flow restriction training in older adults with or susceptible to sarcopenia: A systematic review and meta-analysis

**DOI:** 10.3389/fphys.2022.924614

**Published:** 2022-08-15

**Authors:** Lawrence P. Cahalin, Magno F. Formiga, Brady Anderson, Gerson Cipriano, Edgar D. Hernandez, Johnny Owens, Luke Hughes

**Affiliations:** ^1^ Department of Physical Therapy, Miller School of Medicine, University of Miami, Coral Gables, FL, United States; ^2^ Departamento de Fisioterapia, Faculdade de Medicina, Universidade Federal do Ceará, Fortaleza, CE, Brazil; ^3^ Departamento de Fisioterapia, Universidade de Brasília, Brasília, DF, Brazil; ^4^ Departamento del Movimiento Corporal Humano y sus desórdenes, Universidad Nacional de Colombia, Bogotá, Colombia; ^5^ Owens Recovery Science, San Antonio, TX, United States; ^6^ Department of Sport, Exercise and Rehabilitation, Northumbria University, Northumbria, United Kingdom

**Keywords:** blood flow restriction, vascular occlusion training, aged, elderly, sarcopenia, skeletal muscle function, physical function, functional performance

## Abstract

**Background:** The extent to which exercise training with blood flow restriction (BFR) improves functional performance (FP) in people with sarcopenia remains unclear. We performed a comprehensive search of BFR training in subjects with sarcopenia or susceptible to sarcopenia hoping to perform a systematic review and meta-analysis on the effects of BFR on FP in older adults without medical disorders, but with or susceptible to sarcopenia.

**Methods:** PubMed and the Cochrane library were searched through February 2022. Inclusion criteria were: 1) the study examined older adults (>55 years of age) with or susceptible to sarcopenia and free of overt acute or chronic diseases, 2) there was a random allocation of participants to BFR and active control groups, 3) BFR was the sole intervention difference between the groups, and 4) the study provided post-intervention measures of skeletal muscle and physical function which were either the same or comparable to those included in the revised European Working Group on Sarcopenia in Older People (EWGSOP) diagnostic algorithm.

**Results:** No studies of BFR training in individuals with sarcopenia were found and no study included individuals with FP values below the EWGSOP criteria. However, four studies of BFR training in older adults in which FP was examined were found. BFR training significantly improved the timed up and go (MD = −0.46, z = 2.43, *p* = 0.02), 30-s chair stand (MD = 2.78, z = 3.72, *p* < 0.001), and knee extension strength (standardized MD = 0.5, z = 2.3, *p* = 0.02) in older adults.

**Conclusion:** No studies of BFR exercise appear to have been performed in patients with or suspected sarcopenia based on latest diagnostic criteria. Despite the absence of such studies, BFR training was found to significantly improve the TUG, 30-s chair stand, and knee extension strength in older adults. Studies examining the effects of BFR in subjects below EWGSOP cut-off points are needed.

## Introduction

Sarcopenia is defined as a progressive and generalized skeletal muscle disorder that is associated with increased likelihood of adverse outcomes, including falls, fractures, physical disability, and mortality for which methods to identify and manage it are extremely important and warranted (Cruz-Jentoft et al., 2019). The Strength, Assistance with walking, Rising from a chair, Climbing stairs, and Falls (SARC-F) questionnaire provides a rapid assessment of probable sarcopenia using the above five criteria, each of which are scored from a minimum to maximum level of 0–2 resulting in a maximum total SARC-F score of 10 criteria, with scores ≥4 highlighting the need for further testing (Cruz-Jentoft et al., 2019). Furthermore, the revised European Working Group on Sarcopenia in Older People (EWGSOP) consensus on the definition and diagnosis of sarcopenia published in 2019 provides a framework to classify sarcopenia and the impairments associated with it by identifying cut-points for specific tests and measures (Table 1). (Cruz-Jentoft et al., 2019) Examples of such tests and measures include grip strength, chair stand ability, muscle quantity, and functional performance using gait speed, the short physical performance battery (SPPB), the timed up and go (TUG), and 400-m walk tests. Thus, SARC-F scores and outcomes from the above tests and measures can be used to identify the presence of sarcopenia or susceptibility (likely to be influenced) by sarcopenia.

**TABLE 1:** European working group on sarcopenia in older people cut-off points. (Cruz-Jentoft et al., 2019).

Test	Cut-off points for men	Cut-off points for women	References
Grip strength (kg)	<27	<16	Dodds et al. (2014)
Chair stand (sec)	>15 for five rises	—	Cesari et al. (2009)
Gait speed (m/sec)	≤0.8	—	(Studenski et al., 2011; Cruz-Jentoft et al., 2019)
SPPB (0–12 points)	≤8 points	≤8 points	(Guralnik et al., 1995; Pavasini et al., 2016)
TUG (sec)	≥20	≥20	Bischoff et al. (2003)
400 m walk test	Unable to complete or ≥6 min to complete	Unable to complete or ≥6 min to complete	Newman et al. (2006)

Abbreviations: SPPB, short physical performance battery; TUG, timed up and go.

Management of sarcopenia includes a variety of methods with physical activity, exercise training, and nutritional supplementation consistently identified as effective interventions for sarcopenia. (Negm et al., 2022). Resistance training alone or combined with aerobic exercise appears to be the most effective intervention for sarcopenia (Burton and Sumukadas, 2010; Negm et al., 2022). However, resistance training or aerobic exercise performed at the higher intensities required to elicit optimal physiological adaptations may be difficult for older people with or susceptible to sarcopenia. Furthermore, resistance training and aerobic exercise performed at higher intensities may be associated with a greater risk of injury in a frail population of subjects like many individuals with sarcopenia (Skelton and Mavroeidi, 2018; Di Monaco et al., 2020). Thus, low-load resistance training or aerobic exercise with blood flow restriction (BFR) has been suggested as a potential method to improve skeletal muscle strength and decrease the risk of injury in older people with sarcopenia, which may improve adherence to exercise (Hughes et al., 2017; Beckwée et al., 2019; Conceição and Ugrinowitsch, 2019). In fact, a previous systematic review and meta-analysis of BFR training identified 13 studies in which older adults susceptible to sarcopenia underwent BFR training, with eight of the 13 studies suitable for meta-analysis. The results found a moderate effect (Hedges’ g = 0.523) of low-load BFR training compared to training with the same load without BFR on improving skeletal muscle strength (Hughes et al., 2017). Thus, BFR training may be a practical adjunct to increase strength and potentially improve recovery from strengthening exercise. However, no functional performance (FP) measures were examined in the above meta-analysis, which may provide insight into the degree of susceptibility to sarcopenia and effects of BFR training on FP.

However, a relatively recent systematic review of chronic BFR exercise found that data from 13 studies with a total of 332 participants improved a variety of FP measures with the 30 s sit-to-stand and TUG tests being most improved (Clarkson et al., 2019). In this systematic review, studies of individuals with a variety of different medical conditions were included such as body myositis, end-stage kidney disease, knee injury and knee osteoarthritis. Although individuals with sarcopenia may have one or more of the above disorders, it is important to examine the literature in subjects without medical disorders as this may confound the effect of BFR training on sarcopenia. Additionally, we sought to examine the available BFR literature to better identify the degree of susceptibility or presence of sarcopenia using SARC-F and EWGSOP outcomes. Finally, to fully capture the effects of BFR training in subjects with or susceptible to sarcopenia, we examined previous BFR literature in which BFR training was compared to a non-BFR equivalent exercise training group (i.e., active control group) rather than inactive control or different intensity BFR exercise groups. Thus, the purpose of this study was to perform a systematic review of BFR training in subjects with sarcopenia or susceptible to sarcopenia and to conduct a meta-analysis on the effects of BFR on FP in older adults without medical disorders, but with or susceptible to sarcopenia based on SARC-F and EWGSOP outcomes in whom BFR training was compared to a non-BFR equivalent exercise training group.

## Methods

### Search strategy and data sources

A comprehensive literature review was performed in PubMed and the Cochrane library through February 2022. Supplementary Appendix S1 presents the complete search strategy which was conducted in English and included a mix of terms for the key concepts blood flow restriction, sarcopenia, skeletal muscle and physical function. The reference list of eligible studies was also screened to identify other potentially relevant publications.

### Study selection

A study had to meet the following criteria to be included in the meta-analysis: 1) the study was conducted in older adults (>55 years of age) with or susceptible to sarcopenia and free of overt acute or chronic diseases (since such individuals would likely have poorer FP measures and a greater degree of sarcopenia in whom a less realistic effect of BFR training on FP may result), 2) there was random allocation of study participants to BFR and active control groups, 3) BFR was the sole intervention difference between the groups, and 4) the study provided post-intervention outcome measures of skeletal muscle and physical function, which were either the same or comparable to those included in the revised European Working Group on Sarcopenia in Older People (EWGSOP) diagnostic algorithm. (Cruz-Jentoft et al., 2019). Any studies not meeting these criteria were excluded. Studies were only considered for eligibility if they had been peer reviewed and published prior to the search.

### Data extraction and quality assessment

All included studies were assessed for methodological quality and reporting characteristics using the TESTEX tool to assist with the interpretation of results (Smart et al., 2015). Study quality was assessed *via* the Cohen’s kappa, which revealed that study quality was in complete agreement (k = 1) between coders. Two authors independently read and coded each study for descriptive information including: 1) publication year, 2) gender (1 = only males, 2 = only females, 3 = mixed) and 3) age of the participants in the studies. For both BFR and standard training protocols, the mode of exercise performed and exercise training intensity were coded (1 = walking/treadmill protocol, 2 = resistance training protocol, 3 = other (e.g., functional training); and 1 = low to moderate intensity, 2 = high intensity, respectively). Means, standard deviations, and sample size of post-intervention data for the following outcome measures were obtained and recorded as continuous variables: Timed Up and Go, in seconds taken to complete the test activity; 30-Second Chair Stand, in number of repetitions performed; 6-Minute Walk Test, in distance walked in meters; and the Romberg Test, in seconds the patient was able to stand with eyes closed. Means and standard deviations of post-intervention knee extension strength measures were also recorded as continuous variables when available for supplementary pooled analyses carried out for discussion purposes. Cohen’s kappa determined that the coders were in complete agreement (k = 1). Pearson correlation analysis also demonstrated complete consistency among raters (r = 1).

### Data synthesis and analysis

RevMan, version 5.3 (Cochrane, London, United Kingdom) was used for data analyses. Overall effect estimates were calculated using random-effects models with inverse variance weighting to allow us to address any existing heterogeneity. Either standardized or unstandardized mean differences were computed for each pooled analysis as appropriate along with I^2^ information, representing the percentage of the variability in effect estimates due to heterogeneity. 95% confidence intervals of each study were also calculated. Z scores provided the overall effect of intervention versus control with statistical significance set at a *p*-value < 0.05.

## Results

No studies of BFR training in individuals with sarcopenia meeting our inclusion criteria could be found and no study included individuals with mean ± SD scores (or individual scores when available) of FP below the EWGSOP criteria listed in Table 1. Also, no studies of BFR training in which SARC-F scores were reported could be found.

**FIGURE 1:**
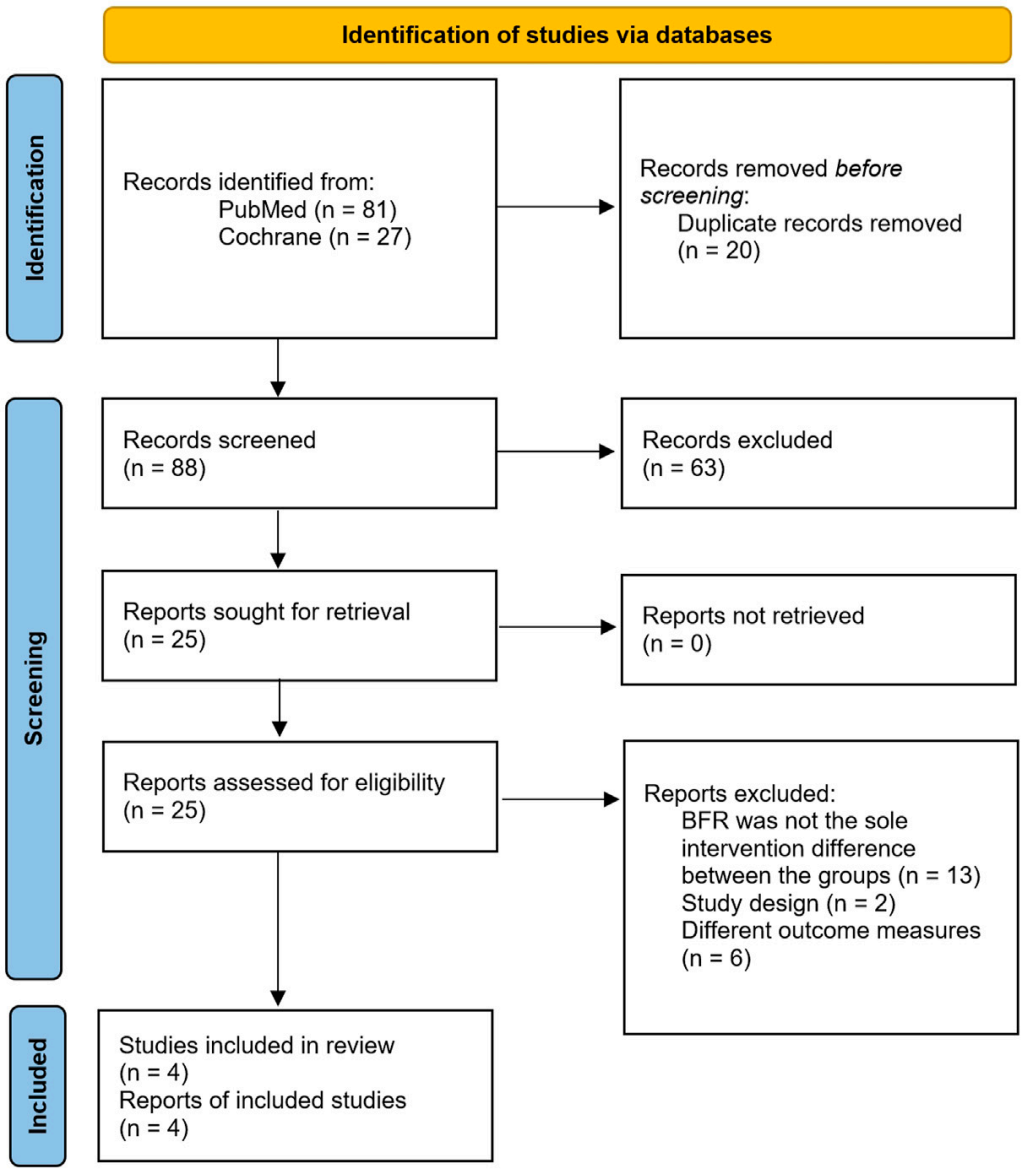
Flow diagram of study selection.

**FIGURE 2:**
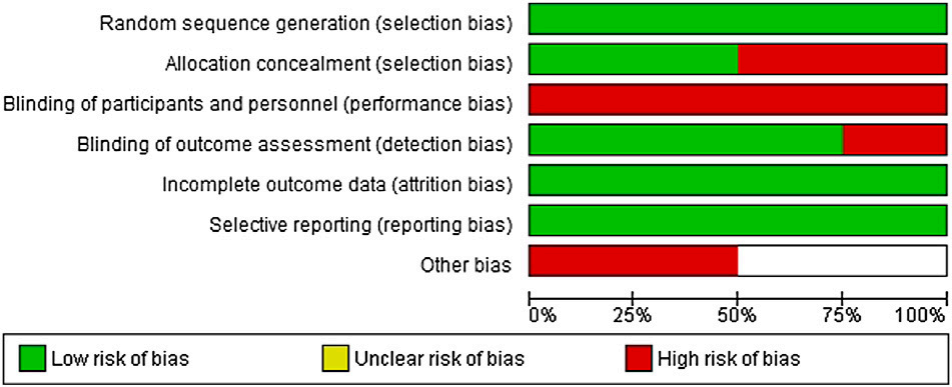
Analysis of risk of bias according to Cochrane Collaboration guidelines.

However, four studies of BFR training in older adults in which FP was examined were found and included the following FP measures: TUG (*n* = 4), (Ozaki et al., 2011; Clarkson et al., 2017; Bigdeli et al., 2020; Kargaran et al., 2021) 30-s chair stand test (*n* = 3), (Ozaki et al., 2011; Clarkson et al., 2017; Kargaran et al., 2021) 6-min walk test (6MWT; *n* = 2), (Clarkson et al., 2017; Kargaran et al., 2021) Romberg balance test (*n* = 2), (Bigdeli et al., 2020; Kargaran et al., 2021) and knee extension strength (*n* = 3) (Ozaki et al., 2011; Bigdeli et al., 2020; Kargaran et al., 2021). A flow diagram of the studies retrieved for the meta-analysis is presented in Figure 1, as per PRISMA reporting guidelines (Page et al., 2021). Table 2 provides an overview of these four studies. The quality of the studies using the TESTEX assessment tool is shown in Table 3, which found that two of the studies had excellent quality and very good reporting characteristics Bigdeli et al. (2020), Kargaran et al. (2021) with the other two studies having moderate to good quality and reporting Clarkson et al. (2017), Ozaki et al. (2011). Risk of bias assessment as per Cochrane Collaboration guidelines is presented in Figure 2.

**TABLE 2:** Overall characteristics of participants per study.

Author (Year)	Population (mean age)	BFR group	Non-BFR equivalent exercise group	Baseline BFR group FP measures and mean score	Baseline Non-BFR equivalent ex. Group FP measures and mean score
Ozaki et al. (2011) ^8^	Older women (66 ± 1 year)	*n* = 10; 20 min of TM walking at 45% HRR, 4x/week for 10 weeks with Kaatsu-Master cuffs placed on the most proximal portion of each leg at AOP of 140–200 mm Hg	*N* = 8; 20 min of TM walking at 45% HRR without BFR, 4x/week for 10 weeks	TUG = 5.0 s 30 s chair stand = 23 knee ext. Torque (nm) Iso = 120 30°/sec = 103 180°/sec = 66	TUG = 4.9 s 30 s chair stand = 24 knee ext. Torque (nm) Iso = 120 30°/sec = 98 180°/sec = 65
Bigdeli et al. (2020) ^11^	Older men (67.7 ± 5.8 years)	*n* = 10; 2–4 sets of 10 reps at 11 FT stations alternating between UE and LE exercise performed at 25%–35% of 1RM with cuffs placed on the proximal extremities at AOP of 50%–70%, 3x/week for 6 weeks with cuffs deflated during 1 min rest periods between sets	*n* = 10; 2–4 sets of 10 reps at 11 FT stations alternating between UE and LE exercise performed at 25%–35% of 1RM without BFR	TUG = 10 s Romberg = 5.5 knee ext. Strength (kg) = 31.7	TUG = 10.9 s Romberg = 4.6 knee ext. Strength (kg) = 31.0
Kargaran et al. (2021) ^9^	Older women (62.9 ± 3.1 yr)	*n* = 8; 20 min of TM walking, 3x/week for 8 weeks at 45% HRR while performing several cognitive tasks with cuffs placed on the proximal LE at AOP of 50% which was increased by 10% every 2 weeks	*N* = 8; 20 min of TM walking, 3x/week for 8 weeks at 45% HRR while performing several cognitive tasks without BFR	TUG = 6.4 s 30 s chair stand = 19.7 6MWT = 530 m Romberg = 6.5 knee ext. Strength (kg) = 19.8	TUG = 7.2 s 30 s chair stand = 18.4 6MWT = 479 m Romberg = 7.4 knee ext. Strength (kg) = 19.6
Clarkson et al. (2017) ^10^	Sedentary older men and women (BFR and non-BFR group age was 69 ± 6 and 70 ± 7 years, respectively)	*n* = 10 (6 men, 4 women); 10 min of walking at 4 km h^−1^ around a 667 m field circuit 4x/week for 6 weeks with cuffs placed on the most proximal portion of each leg at AOP of 60%	*n* = 9 (5 men, 4 women); 10 min of walking at 4 km h^−1^ around a 667 m field circuit 4x/week for 6 weeks	TUG = 6.6 s 30 s chair stand = 14.5 6MWT = 505 m	TUG = 6.75 s 30 s chair stand = 14.9 6MWT = 528 m

Abbreviations: 1-RM, One-repetition maximum; 6MWT, Six-minute walk test; AOP, arterial occlusion pressure; BFR, blood flow restriction; FP, functional performance; FT, functional training; HRR, heart rate reserve; LE, lower extremity; TM, treadmill; TUG, timed up and go; UE, upper extremity.

**TABLE 3:** TESTEX assessment of the quality and reporting of included randomized controlled trials.

	Study quality criterion						Study reporting criterion											
Study	1	2	3	4	5	Total	6a	6b	6c	7	8a	8b	9	10	11	12	Total	Overall Total
Bigdeli et al. (2020)	1	1	1	1	1	5	1	0	0	1	1	1	1	0	1	1	7	12
Clarkson et al. (2017)	1	1	0	1	0	3	1	0	0	1	1	1	1	1	0	1	7	10
Kargaran et al. (2021)	1	1	1	1	1	5	1	0	1	1	1	1	1	0	0	1	7	12
Ozaki et al. (2011)	1	1	0	1	1	4	1	0	0	1	1	1	1	0	0	1	6	10

Two of the studies included only older women, one study included only older men, and one study included both men and women who were older. The age range of subjects in the studies was from 62.9 ± 3.1 to 70 ± 7 years (Ozaki et al., 2011; Clarkson et al., 2017; Bigdeli et al., 2020; Kargaran et al., 2021). The number of subjects in the BFR and non-BFR groups was relatively well matched, with two studies having the same number of subjects and the other two studies differing by one and two subjects per group. The four studies included a total of 73 individuals of whom 57.5% were women. (Ozaki et al., 2011; Clarkson et al., 2017; Bigdeli et al., 2020; Kargaran et al., 2021).

Treadmill walking with and without BFR was performed in two of the studies at the same intensity (45% of HRR) (Ozaki et al., 2011; Kargaran et al., 2021) with one of the studies imposing cognitive-tasks while walking (Kargaran et al., 2021). The study imposing cognitive tasks while walking was performed for 20 min, 3x/week for 8 weeks, (Kargaran et al., 2021) and the other treadmill walking study was also performed for 20 min, but with a slightly greater frequency and duration (4x/week for 10 weeks) (Ozaki et al., 2011). A third study also used walking as the mode of exercise with and without BFR which was done around a 667 m field circuit for 10 min at 4 km h^−1^, 4x/week for 6 weeks (Clarkson et al., 2017). The fourth study performed functional training (FT) exercises with and without BFR, which included 2–4 sets of 10 reps at 11 FT stations alternating between UE and LE exercise performed at 25%–35% of 1RM. (Bigdeli et al., 2020).

The only EWGSOP FP measure for sarcopenia that was reported in the included studies was the TUG (Cruz-Jentoft et al., 2019). None of the participants enrolled in the four studies included in this meta-analysis approached the cut-off score of ≥20 s with participants in three of four studies completing the TUG in less than 10 s, (Ozaki et al., 2011; Clarkson et al., 2017; Kargaran et al., 2021) and in the other study the BFR and non-BFR group participants completed the TUG in 10 and 10.9 s, respectively (Bigdeli et al., 2020). No reports of adverse events during or after BFR or non-BFR equivalent exercise were found in any of the studies (Ozaki et al., 2011; Clarkson et al., 2017; Bigdeli et al., 2020; Kargaran et al., 2021).

**FIGURE 3:**
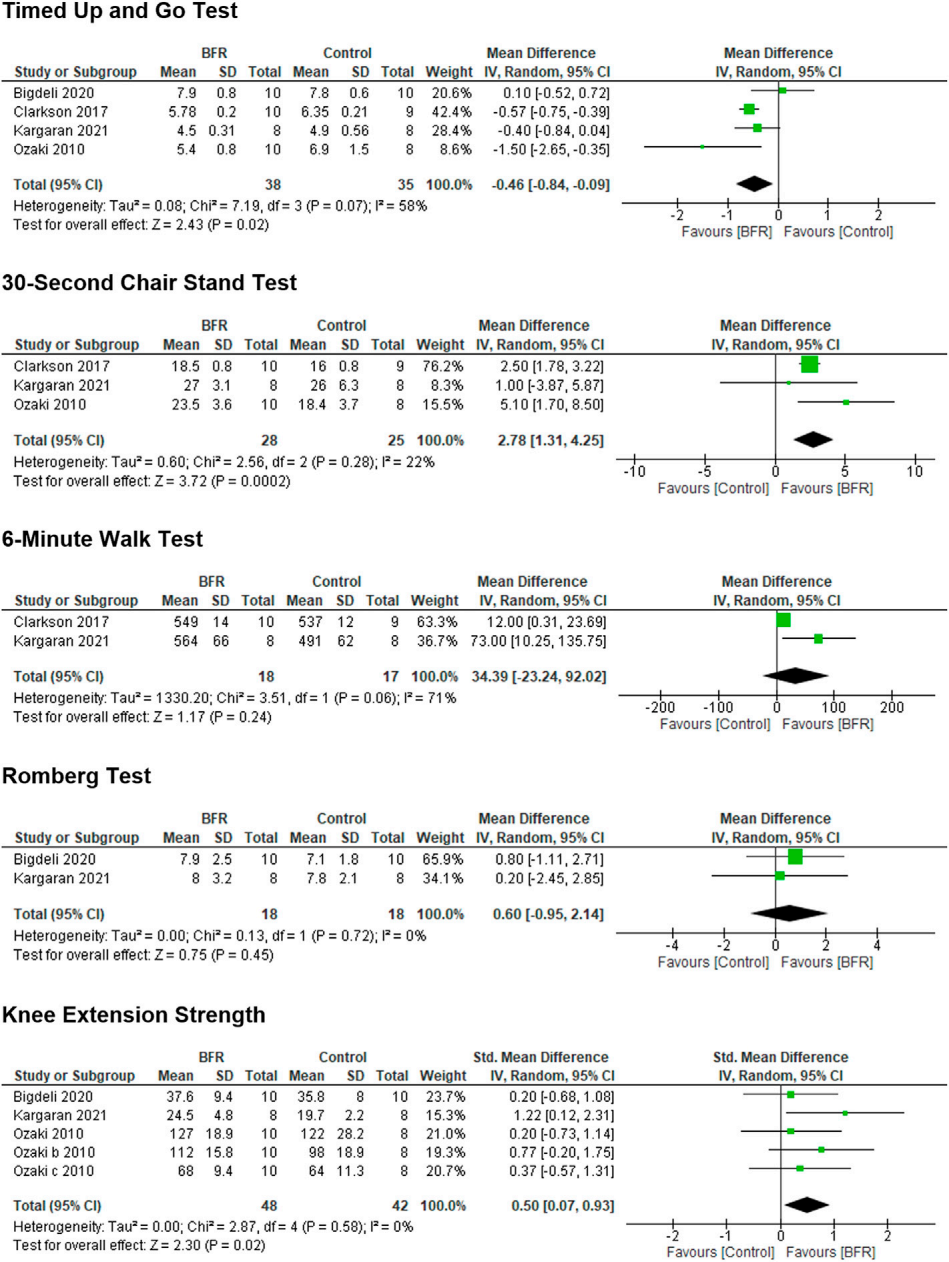
Forest plots showing the effects of blood flow restriction training on different functional performance measures in older adults.

The Forest Plots and results of the meta-analyses for the TUG, 30-s chair stand, 6MWT, Romberg balance test, and knee extension strength are shown in Figure 3, in which BFR training was found to significantly improve the TUG, 30-s chair stand, and knee extension strength in older adults. BFR training had no significant effect on the 6MWT or Romberg balance test, but the results favored BFR training compared to a non-BFR equivalent exercise training group (Figure 3). The degree of heterogeneity reflected by the I^2^ values in the 30-s chair stand test, knee extension strength, and Romberg balance test was low (16%, 0%, and 0%, respectively), while that of the TUG was modest (58%), and the 6MWT high (71%).

## Discussion

The results of a comprehensive search for studies examining the effects of BFR training in older adults with or susceptible to sarcopenia using SARC-F and EWGSOP outcomes was disappointing. No study of BFR training could be found using the SARC-F criteria and the only EWGSOP FP measure for sarcopenia that could be used was the TUG. None of the participants enrolled in the four studies included in this meta-analysis approached the cut-off score of ≥20 s. In view of the above results, studies examining the effects of BFR on FP in subjects with sarcopenia are needed. Nonetheless, this is the first meta-analysis to examine the effects of BFR training on FP in older adults without medical disorders and found that no studies of BFR appear to have been performed in patients with sarcopenia or suspected sarcopenia based on SARC-F and EWGSOP outcomes.

Despite the absence of studies examining the effects of BFR exercise in patients with or susceptible to sarcopenia, BFR training was found to significantly improve the TUG, 30-s chair stand, and knee extension strength compared to exercise training without BFR in older adults. These are important findings in regards to the FP of older adults and likely to the FP of individuals with sarcopenia, who in view of the EWGSOP criteria, will have poorer baseline FP, (Cruz-Jentoft et al., 2019) and may have even greater improvements in these and other FP measures. Also, although knee extension strength may not be a true FP measure, it is significantly correlated to a variety of FP measures, including gait speed, chair stand, and balance (Cai et al., 2022). Furthermore, isokinetic knee extension and flexion strength appear to have the ability to identify sarcopenia (Steffl and Stastny, 2020; kis et al., 2022). Several review articles have presented the rationale for BFR being an effective non-pharmacological treatment of sarcopenia, (Beckwée et al., 2019; Conceição and Ugrinowitsch, 2019) but research focused on the effects of BFR training in patients with sarcopenia is lacking and thus identifies the need for future investigation given the results of this study. Thus, a call for action for research and research funding to support and perform studies on BFR training in subjects with sarcopenia is desperately needed in view of the aging population in the United States (2020 Profile of Older Americans, 2021; von Elm et al., 2008; Bischoff et al., 2003) and the globe (World Population Ageing 2019: Highlights, 2019; Newman et al., 2006) as well as the improvements in FP observed in this study. Future research must ensure to implement BFR according to current understanding of optimal parameters, such as training being individualized to limb occlusion pressure, appropriate loading and progression (Patterson et al., 2019). While research in individuals with sarcopenia is warranted, it is important that further investigations follow best practice as methodological heterogeneity will limit the formulation of accurate and informative conclusions on training effectiveness and safety.

One case report of BFR training in a 91-year old sedentary man diagnosed with sarcopenia [appendicular skeletal muscle mass (ASM) of 7.10 kg/m^2^) was found in which the subject presented with exhaustion, lower-limb weakness, hypertension, and a history of multiple falls (Lopes et al., 2019). Three months of low-intensity upper and lower extremity resistance training (3 sets of 10 repetitions at 30% of one RM for elbow flexion and extension, knee extension, and leg press) were initially performed which was followed by 1 month of inactivity during, which the subject was asked to maintain instrumental activities of daily living (ADLs) and avoid any changes in his routine. Following the 1 month of inactivity, the subject performed 8 weeks of BFR training at the same intensity while exercising with the same number of sets, repetitions, and muscle groups (Lopes et al., 2019). Protein supplementation was provided to the subject after low-intensity resistance training with and without BFR (Lopes et al., 2019). The results of low-intensity resistance training with BFR on body composition, sarcopenia cut-points, and strength were greater than that observed with low-intensity resistance training without BFR. For example, low-intensity resistance training without BFR resulted in 2.7% decreases in ASM and total skeletal muscle mass (SMM), but low-intensity resistance training with BFR produced 2.3 and 2.1% increases in these same respective measures (Lopes et al., 2019). Handgrip strength was found to decrease 3.4% after low-intensity resistance training without BFR, but increased 17.9% after low-intensity resistance training with BFR (Lopes et al., 2019). Furthermore, isokinetic knee extension peak torque, total work, and work fatigue decreased after low-intensity resistance training without BFR (8.8%–20.4%), but increased after low-intensity resistance training with BFR (1.5%–27.5%). Additionally, interleukin-6 (IL-6) and insulin-like growth factor-1 (IGF-1) were improved, but endothelin-1 and oxidative stress increased with less endothelial vasoreactivity after low-intensity resistance training with BFR (Lopes et al., 2019). In view of the above findings in a single case subject, BFR training has the potential to improve key pathophysiological manifestations of sarcopenia, but further investigation of its effects on oxidative stress and endothelial function is needed.

Two of the studies included in this meta-analysis examined blood markers indirectly related to oxidative stress and endothelial function and directly related to neuromuscular activity (Bigdeli et al., 2020; Kargaran et al. (2021) examined the effects of dual-task treadmill walking with and without BFR on brain-derived neurotrophic factor (BDNF), procollagen III N-terminal peptide (P3NP), and C-terminal Agrin (CAF), and found significant increases in BDNF after 8 weeks in both dual-task walking with and without BFR which was not observed in the control group that performed everyday activities, but without dual-tasks. (Kargaran et al., 2021) Furthermore, the CAF concentration in the dual-task walking with BFR group was significantly lower than that observed in the dual-task walking without BFR or control groups. Finally, only the dual-task walking group was found to have a significant increase in the level of P3NP after the 8-weeks study period (Kargaran et al., 2021). The increase in P3NP is suggestive of a greater anabolic response while the decrease in CAF suggests less neuromuscular junction remodeling, degradation, and muscle wasting. Bigdeli et al. (2020) also examined CAF and P3NP levels before and after functional training with and without BFR and found results similar to Kargaran et al. (2021) in that CAF levels were lower after 6 weeks of functional training with BFR than after functional training without BFR, and were significantly lower than levels in a control group which maintained ADLs (Bigdeli et al., 2020). Although Bigdeli found no significant difference in P3NP levels among groups, the decrease in P3NP observed in all groups was less in the functional training with BFR group (Bigdeli et al., 2020). In view of the above, we performed an additional meta-analysis on the CAF and P3NP results from these two studies and found a significant decrease in CAF after exercise with BFR compared to exercise without BFR [Std. Mean Diff = −0.76, (95% CI: 1.44, −0.07); Z = 2.17; *p* = 0.03; I^2^ = 0%] and no effect on P3NP [Std. Mean Diff = 0.14, (95% CI: 0.90, 1.19); Z = 0.27; *p* = 0.79; I^2^ = 58%] (Bigdeli et al., 2020; Kargaran et al., 2021). Thus, in view of the above results, BFR exercise has the potential to decrease CAF levels suggesting less neuromuscular junction remodeling, degradation, and muscle wasting all of which would be beneficial for the FP of subjects with sarcopenia.

One final note related to the above blood markers and FP is that Bigdeli et al. (2020) found significant negative correlations between the level of CAF and knee extension, chest press, and static balance and significant positive correlations between the level of P3NP and chest press (Bigdeli et al., 2020). Similarly, Kargaran et al. (2021) found a significant negative correlation between CAF level and leg skeletal muscle quality and a significant positive correlation between P3NP level and leg skeletal muscle quality only in the BFR exercise group (Kargaran et al., 2021). Kargaran also found that BDNF level was significantly correlated to the Mini-Mental State Examination in all groups. Although these findings are encouraging for patients with sarcopenia, further examination of BFR training on the above blood markers and their relationship to FP and cognition in older adults with sarcopenia is needed.

Several limitations of this meta-analysis with systematic review exist, including a small number of studies and number of subjects included in the analyses as well as a small number of studies examining the level of CAF and P3NP. Although the inclusion of only studies in which healthy subjects without medical disorders were enrolled and BFR training was compared to a non-BFR equivalent exercise training group limited the number of studies included in our analyses, we believe it is a strength of the study. The finding that no studies of BFR exercise in subjects with sarcopenia or suspected sarcopenia exist is worrisome and identifies the need for research focus and funding to examine the effects of BFR exercise on skeletal muscle strength, quantity, quality, and FP as outlined by the EWGSOP (Cruz-Jentoft et al., 2019). The examination of BFR exercise compared to a non-BFR equivalent exercise in subjects below the EWGSOP cut-off points is needed and in view of the results of this meta-analysis and systematic review may identify an important non-pharmacologic intervention for sarcopenia.

## Conclusion

No studies of BFR exercise appear to have been performed in patients with sarcopenia or suspected sarcopenia based on SARC-F and EWGSOP outcomes. However, despite the absence of studies examining the effects of BFR exercise in patients with or susceptible to sarcopenia, BFR training was found to significantly improve the TUG, 30-s chair stand, and knee extension strength in older adults making BFR exercise a practical adjunct in the management of subjects with sarcopenia. The only EWGSOP FP cut-off point for sarcopenia that could be used was the TUG and one of the participants enrolled in the four studies included in this meta-analysis approached the EWGSOP cut-off score of ≥20 s with participants in three of four studies completing the TUG in less than 10 s, (Ozaki et al., 2011; Clarkson et al., 2017; Kargaran et al., 2021) and in the other study, the BFR and non-BFR group participants completed the TUG in 10 and 10.9 s, respectively (Bigdeli et al., 2020). In view of the above results, studies examining the effects of BFR on FP as well as skeletal muscle strength, quantity, and quality as outlined in the EWGSOP consensus in subjects with sarcopenia are needed (Cruz-Jentoft et al., 2019). Furthermore, further investigation of isokinetic testing appears warranted in view of the significant improvement in knee extension strength observed in this study as well as other literature highlighting its potential role in identifying sarcopenia. (Steffl and Stastny, 2020; kis et al., 2022). The significant improvements in FP of older adults observed in this study is important especially in view of the EWGSOP criteria in which older adults with sarcopenia are likely to have poorer baseline FP with the potential for even greater improvements in these and other FP measures.

## Data Availability

The original contributions presented in the study are included in the article/Supplementary Material, further inquiries can be directed to the corresponding author.
