# Supplementary material for: A call to action for blood flow restriction training in older adults with or susceptible to sarcopenia: A systematic review and meta-analysis
Source: Front Physiol. 2022 Aug 15;13:924614. doi: 10.3389/fphys.2022.924614 (PMC9421943; doi:10.3389/fphys.2022.924614)
Supplement: Supplementary file 1 [file Table1.docx]

**APPENDIX 1**

The following clinical question and its respective concept map were used to develop the search strategy: What is the effectiveness of blood flow restriction training (BFR) compared to no-BFR on muscle mass, muscle strength and functional performance in older adults with or susceptible to sarcopenia?

| P: Older adults with or susceptible to sarcopenia | I: BFR | C: No-BFR | O: Muscle mass, muscle strength, functional performance |
| --- | --- | --- | --- |
| Sarcopenia (MeSH) | Resistance Training (MeSH) |  | Muscle, Skeletal (MeSH) |
| Aged (MeSH) | Exercise Therapy (MeSH) |  | Muscle Mass (Keyword) |
|  | Blood Flow Restriction (Keyword) |  | Muscle Strength (MeSH) |
|  | Kaatsu Training (Keyword)  Vascular Occlusion (Keyword) |  | Physical Fitness (MeSH) |

**PubMed search:** (((sarcopenia[MeSH Terms]) OR (aged[MeSH Terms])) AND ((((((resistance training[MeSH Terms]) OR (exercise therapy [MeSH Terms])) AND ("blood flow restriction")) OR ("kaatsu training")) OR ("kaatsu")) OR ("vascular occlusion"))) AND ((((muscle, skeletal[MeSH Terms]) OR ("muscle mass")) OR (muscle strength[MeSH Terms])) OR (physical fitness[MeSH Terms]))

74 records were identified with the above search.

**Cochrane search:**#1 MeSH descriptor: [Sarcopenia]
#2 MeSH descriptor: [Aged]
#3 #1 or #2
#4 MeSH descriptor: [Resistance Training]
#5 MeSH descriptor: [Exercise Therapy]
#6 (Blood Flow Restriction):ti,ab,kw
#7 (KAATSU Training):ti,ab,kw
#8 (Vascular Occlusion):ti,ab,kw
#9 #4 or #5
#10 #6 or #7 or #8
#11 #9 and #10
#12 MeSH descriptor: [Muscle, Skeletal]
#13 (Muscle Mass):ti,ab,kw
#14 MeSH descriptor: [Muscle Strength]
#15 MeSH descriptor: [Physical Fitness]
#16 #12 or #13 or #14 or #15
#17 #3 and #11 and #16
